# A modeling study of cortical waves in primary auditory cortex

**DOI:** 10.1186/1471-2202-14-S1-P23

**Published:** 2013-07-08

**Authors:** David Beeman

**Affiliations:** 1 Department of Electrical, Computer, and Energy Engineering, University of Colorado, Boulder, CO 80309, USA

## 

Cortical waves have been observed in many cortical areas, including the primary auditory cortex (AI). The thalamorecipient layer (IV) of AI is an ideal test-bed for the study of cortical waves because the inputs from the thalamus are arranged tonotopically along an axis. Wave propagation along this axis is essentially one-dimensional. Thus, a biologically realistic model of a piece of this area can act like a 'ripple-tank' used for the study of one-dimensional wave motion in physics.

**Figure 1:**
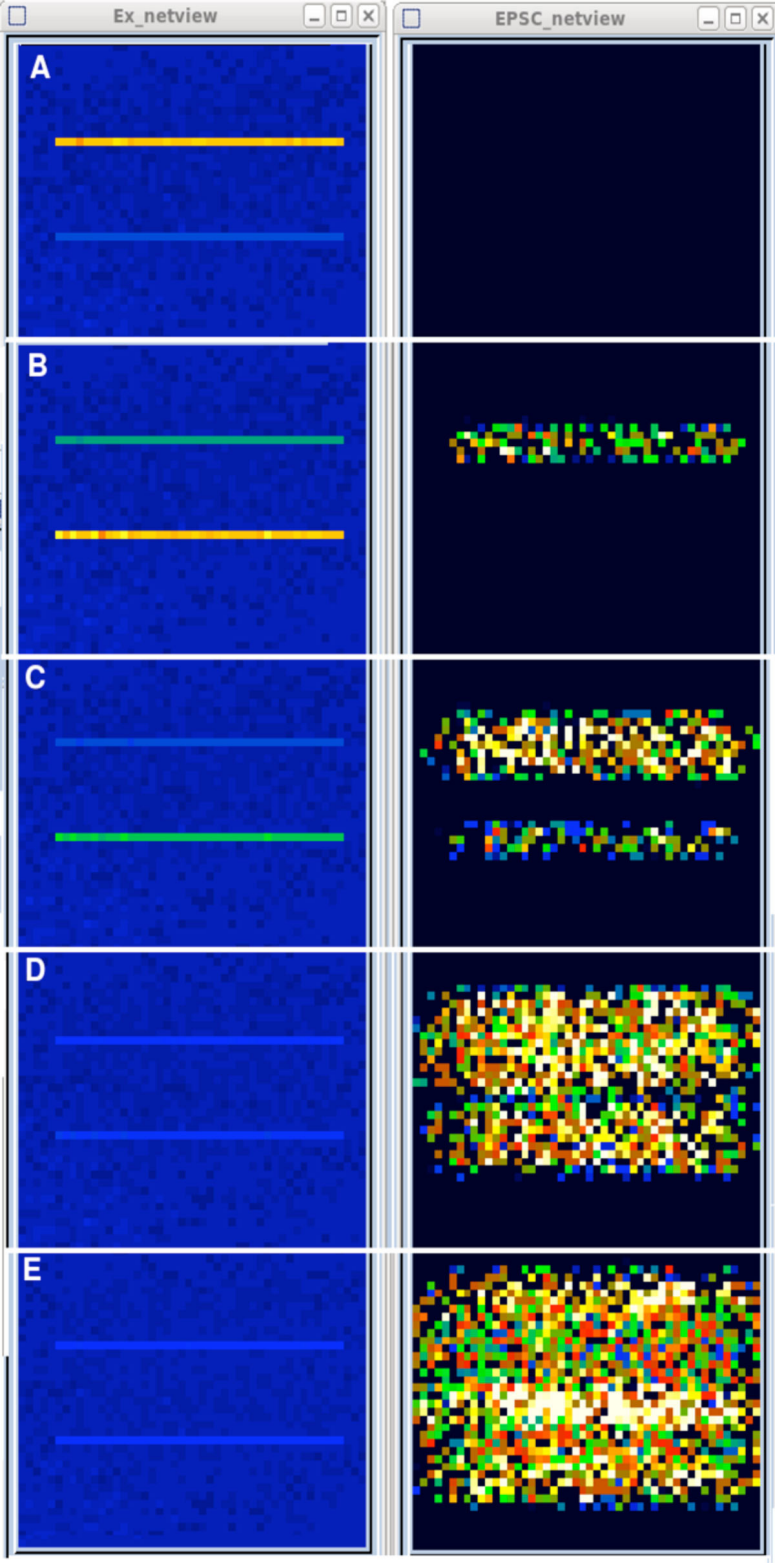
**Frames **(A-E) **show successive intervals in the development and interference **(E) **of cortical waves from a two-tone input**. Membrane potential is shown on the left and excitatory post-synaptic currents on the right.

The simulations described here were implemented with the GENESIS 2.3 and 3 (G-3) simulators [1]. The primary objective of this modeling study was to determine the effects of axonal conduction velocity (often neglected, but significant), as well as synaptic time constants, on the ability of such a network to create and propagate cortical waves. Figure 1 illustrates that, using parameters that are within the range of commonly measured experimental values, such propagation (A-D), and even constructive interference (E), is possible. The model is also being used to study the interaction between single and two-tone input and normal background activity, and the effects of synaptic depression from thalamic inputs. The simulation scripts have the additional purpose of serving as tutorial examples for the construction of cortical networks with GENESIS. The present model has fostered the development of the G-3 Python network analysis and visualization tools used in this study [2]. Full simulation scripts and documentation with tutorials for extending them to a more complete model of auditory cortex will be available on the http://genesis-sim.org web site.

AI has been less well studied than other neocortical areas, and any complete model will contain an enormous space of poorly-quantified parameters to be explored. In order to simplify the model enough to analyze, it was limited to a population of pyramidal cells and a smaller population of inhibitory basket cells. Inputs from other cortical layers were crudely represented by Poisson-distributed random inputs to provide background levels of firing for the two populations that are in agreement with measured values. The components within this simple model are represented realistically, with parameters constrained by experimental measurements. The multi-compartmental spiking neurons have firing patterns representative of pyramidal and basket cells in the auditory cortex. Synaptic inputs occur at appropriate locations on the dendrites. The distance dependence of the connection probabilities for the four types of connections was fit to experimental measurements. It has equivalent ranges for excitatory and inhibitory connections, unlike many models that account for lateral inhibition by assuming a greater range for inhibitory connections. Although the thalamic input model provides for more realistic inputs, the example shown in Figure 1 used single row excitations of spike trains at 220 and 440 Hz in the absence of background input.
